# Dynamic detection of three-dimensional crop phenotypes based on a consumer-grade RGB-D camera

**DOI:** 10.3389/fpls.2023.1097725

**Published:** 2023-01-27

**Authors:** Peng Song, Zhengda Li, Meng Yang, Yang Shao, Zhen Pu, Wanneng Yang, Ruifang Zhai

**Affiliations:** ^1^ National Key Laboratory of Crop Genetic Improvement, National Center of Plant Gene Research (Wuhan), Huazhong Agricultural University, Wuhan, China; ^2^ College of Informatics, Huazhong Agricultural University, Wuhan, China

**Keywords:** field crop phenotype, RGB-D camera, point cloud segmentation, dynamic 3D reconstruction, crop seedling detection

## Abstract

**Introduction:**

Nondestructive detection of crop phenotypic traits in the field is very important for crop breeding. Ground-based mobile platforms equipped with sensors can efficiently and accurately obtain crop phenotypic traits. In this study, we propose a dynamic 3D data acquisition method in the field suitable for various crops by using a consumer-grade RGB-D camera installed on a ground-based movable platform, which can collect RGB images as well as depth images of crop canopy sequences dynamically.

**Methods:**

A scale-invariant feature transform (SIFT) operator was used to detect adjacent date frames acquired by the RGB-D camera to calculate the point cloud alignment coarse matching matrix and the displacement distance of adjacent images. The data frames used for point cloud matching were selected according to the calculated displacement distance. Then, the colored ICP (iterative closest point) algorithm was used to determine the fine matching matrix and generate point clouds of the crop row. The clustering method was applied to segment the point cloud of each plant from the crop row point cloud, and 3D phenotypic traits, including plant height, leaf area and projected area of individual plants, were measured.

**Results and Discussion:**

We compared the effects of LIDAR and image-based 3D reconstruction methods, and experiments were carried out on corn, tobacco, cottons and Bletilla striata in the seedling stage. The results show that the measurements of the plant height (R²= 0.9~0.96, RSME = 0.015~0.023 m), leaf area (R²= 0.8~0.86, RSME = 0.0011~0.0041 *m* ^2^ ) and projected area (R² = 0.96~0.99) have strong correlations with the manual measurement results. Additionally, 3D reconstruction results with different moving speeds and times throughout the day and in different scenes were also verified. The results show that the method can be applied to dynamic detection with a moving speed up to 0.6 m/s and can achieve acceptable detection results in the daytime, as well as at night. Thus, the proposed method can improve the efficiency of individual crop 3D point cloud data extraction with acceptable accuracy, which is a feasible solution for crop seedling 3D phenotyping outdoors.

## 1 Introduction

Crop phenotyping has become a bottleneck restricting crop breeding and functional genomics study since the development of sequencing technology (Yang et al., 2020). To resolve the shortcomings of traditional crop phenotype identification methods, which are labor intensive, time-consuming and frequently destructive to plants, a number of intelligent and high-throughput phenotyping platforms have been developed to investigate crop phenotypic traits (Song et al., 2021). Compared with the laboratory environment, the outdoor environment is much more variable. Because the vast majority of crops are grown in the field, crop phenotyping in the outdoor environment remains one of the major focuses for researchers. Since ground-based phenotyping platforms can obtain crop phenotypic data outdoors with high accuracy by carrying multiple sensors, researchers have developed fixed, as well as mobile ground-based platforms for crop phenotyping (Kirchgessner et al., 2017; Virlet et al., 2017). The disadvantage of a fixed platform is that the system can only cover a limited area, which restricts the application. However, sensors mounted on a moveable platform could overcome these issues (Qiu et al., 2019).

Among the various phenotypic traits of crops, morphological and structural phenotypes can directly indicate plant growth. Continuous measurements of morphological and structural phenotypes play an important role in contributing to crop functional gene selection (Wang et al., 2018). Compared with 2D imaging, 3D imaging techniques allow point cloud acquisition of plants, from which many more spatial and volumetric traits during plant growth can be calculated accurately (Wu et al., 2022). In fact, 3D imaging technologies are the most popular methods to analyze the structure of plants and organs (Ghahremani et al., 2021). Current 3D imaging techniques mainly include image-based, laser scanning-based, and depth camera-based techniques.

The image-based reconstruction technique mainly uses the structure from motion (SfM) algorithm, in which a 3D point cloud is reconstructed by pairing relative feature points extracted from series 2D images of a target. To reconstruct plants, such as wheat seedlings, cucumber, pepper and eggplant, 60 images taken around each plant were needed for smaller plants, and 80 images were needed for taller plants to reconstruct the point cloud stability (Hui et al., 2018). For example, Nguyen built a 3D imaging system using ten cameras, a structured light system, and software algorithms (Nguyen et al., 2015). Approximately 100 images around the tomato plant were taken for individual tomato plant 3D point cloud reconstruction (Wang et al., 2022). Image-based 3D imaging approaches can provide detailed point clouds with inexpensive imaging devices. Nevertheless, images must be taken from multiple cameras or taken at a high frequency on a movable platform to obtain sufficient overlaps for photogrammetry (An et al., 2017).

Light detection and ranging (LiDAR) devices are the main sensors used for laser scanning-based methods. LiDAR can record the spatial coordinates (XYZ) and intensity information of a target by measuring the distance between the sensor and the target with a laser and analyzing the time of flight (ToF) (Sun et al., 2018). Although the spatial resolution of the 3D model produced by LiDAR is not as dense as those obtained by image-based methods, it is sufficient for extracting most plant morphologic traits (Zhu et al., 2021). As an active sensor, LiDAR can operate regardless of illumination conditions, and 3D lasers were considered to have lower throughput for 1-2 minutes were required to collect data at each plot (Virlet et al., 2017).

Depth camera-based methods have been proposed for crop 3D phenotyping in recent years. RGB-D sensors, such as Microsoft Kinect and Intel Realsense cameras, have been widely used due to their low cost and ease of integration (Paulus, 2019).In addition to the three color channels (RGB – Red, Green, Blue), RGB-D sensors provide a depth channel (D), measuring the distance from the sensor to the point in the RGB image with which the length and width of a stem or the size of a fruit, can be estimated. Compared with image-based and laser scanning-based methods, depth camera-based methods are far superior in terms of data acquisition and reconstruction speed (Martinez-Guanter et al., 2019). The feasibility of crop point clouds reconstructed by depth cameras to obtain information on crop phenotypic traits has been demonstrated indoors, and the Kinect family of products has been used more extensively in controlled environments such as greenhouses (Milella et al., 2019). Soybean and leafy vegetables were reconstructed and showed high accuracy in predicting the fresh weight of plants (Hu et al., 2018; Ma et al., 2022). Devices mounted on outdoor mobile platforms typically used shading devices or record with low light to avoid inaccuracies in depth camera detection (Andújar et al., 2016) and have been validated by researchers for detecting corn orientation, plant height, and leaf tilt information (Bao et al., 2019; Qiu et al., 2022).

Together with sensors, robotic systems will substantially enhance the capacity, coverage, repeatability, and cost-effectiveness of plant phenotyping. Dynamic data collection with robotic movement can greatly improve the efficiency of crop phenotyping, especially in the field. Mueller-Sim et al. (Mueller-Sim et al., 2017) established a robot equipped with a custom depth camera and a manipulator capable of measuring sorghum stalks while moving between crop rows. An RGB-D camera was installed on GPhenoVision, a mobile platform based on a high-clearance tractor for field-based high-throughput phenotyping, for cotton 3D data acquisition. The results showed that a Kinect camera with a shadowing device could provide accurate raw data for plant morphological traits from canopies in the field (Jiang et al., 2018). Compared with Kinect, the Realsense sensor has also shown strong capabilities in reconstructing point clouds indoors (Milella et al., 2019), and its depth information is more accurate and has a much higher fill rate than the Kinect camera for outdoor plant detection (Vit and Shani, 2018). Generally, the commonly used 3D sensors for phenotyping robots include stereo vision cameras and ToF (time of flight) cameras (Atefi et al., 2021). The reason for this would be that these sensors provide color information along with depth information. Thus, the plant can be effectively segmented, and the traits can be effectively measured.

To efficiently and accurately acquire 3D phenotypic traits of individual plants in an outdoor environment, it is necessary to collect multiple frames of data during the movement of mobile platforms and reconstruct the data continuously. In this study, a method of dynamic detection of individual plant phenotypic traits in a row from the top in an outside environment based on a consumer-grade RGB-D sensor is proposed. Using a Realsense camera equipped on a mobile platform to acquire color and depth information of crops from the top, the specific objectives were to (1) dynamically acquire and verify the robustness of the reconstructed plant point cloud data within different conditions and (2) segment individual plant point clouds to obtain plant phenotypic traits and verify the accuracy of the point cloud reconstruction method.

## 2 Material and methods

An RGB-D sensor was installed on an automatic movable platform for 3D crop reconstruction. Depth information and RGB images were continuously acquired during the movement of the platform to reconstruct the point cloud of each frame. To reconstruct the point clouds of crop rows completely and accurately, it is necessary to match the adjacent point cloud during the movement process. In this study, scale-invariant feature transform (SIFT) was used to pick up the features for matching and to calculate the displacement of homonymous points in the neighboring image continuously.

**Figure 1:**
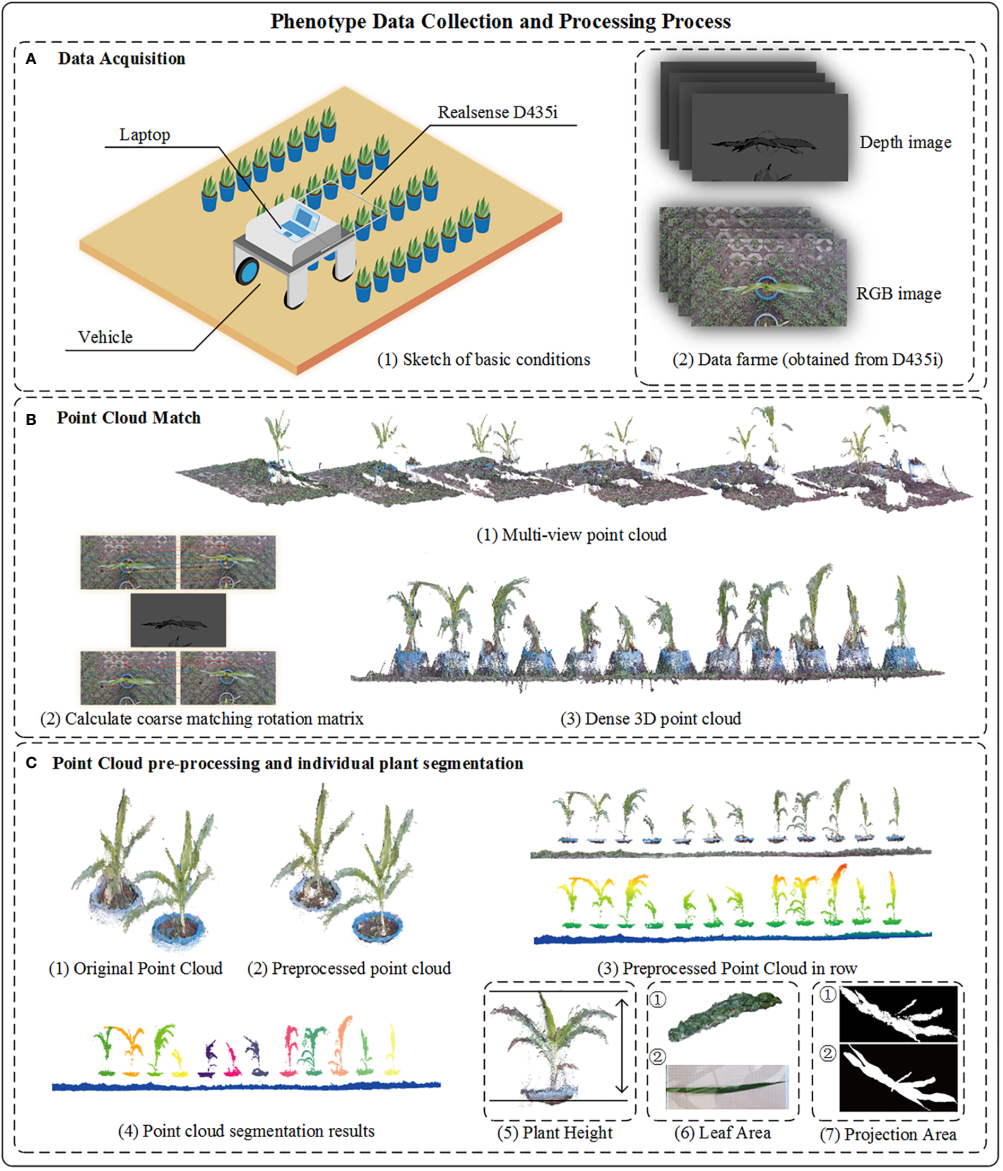
Overall process of crop 3D reconstruction and phenotyping. **(A-1)** The sketch of data acquisition. **(A-2)** Images capture from Realsense D435i, the original of depth image was saved as unit16 format, we have enhanced the depth image to facilitate readers’ understanding in the figure. **(B-1)** Multi-view point cloud reconstruction from sequentially depth and RGB image. **(B-2)** The coarse rotation matrix calculate from adjacent image. **(B-3)** Result of fused point cloud captured in row. **(C-1)** Part of original point cloud in row. **(C-2)** Point cloud with preprocessed method. **(C-3)** Crop point cloud in row. **(C-4)** Results of individual plant by cluster method. **(C-5)** The plant height is from the point cloud plane to the top point. **(C-6)** ① The surface reconstruction result. ② Image of crop leaf. **(C-7)** Binary image of crop projection area ① view of maize point cloud ② view of maize top image.

When the platform moves forward with different speeds, the displacement of homonymous points detected by SIFT in neighboring images on both the X and Y axes of the platform will be calculated based on which data frames of the RGB-D sensor will be selected for crop row point cloud reconstruction. Point clouds of the selected frame were rotated through the coarse matching matrix, which was computed from displacement on the X and Y axes, and then the colored iterative closest point (Colored_ICP) algorithm was used to calculate the fine matching matrix between two point cloud frames. Finally, the discrete points in the point cloud were processed using filters to obtain a high-quality point cloud. The point cloud of each plant was segmented to obtain the height, projected area, and partial leaf area of each plant. **Figure 1** shows the data collection and processing procedure of crop 3D phenotypic traits.

### 2.1 Data acquisition

Data acquisition was performed by a movable platform with an independent four-wheel drive developed in our laboratory. The platform is capable of moving automatically with an integrated visual, inertial guidance and GPS navigation solution at various speeds in the field environment, up to a maximum speed of 1.2 m/s. A RGB-D camera (INTEL RealSense D435i, Intel Corporation, Santa Clara, CA, USA) sells for about $299 was equipped on the platform to perform data acquisition.

**Figure 2:**
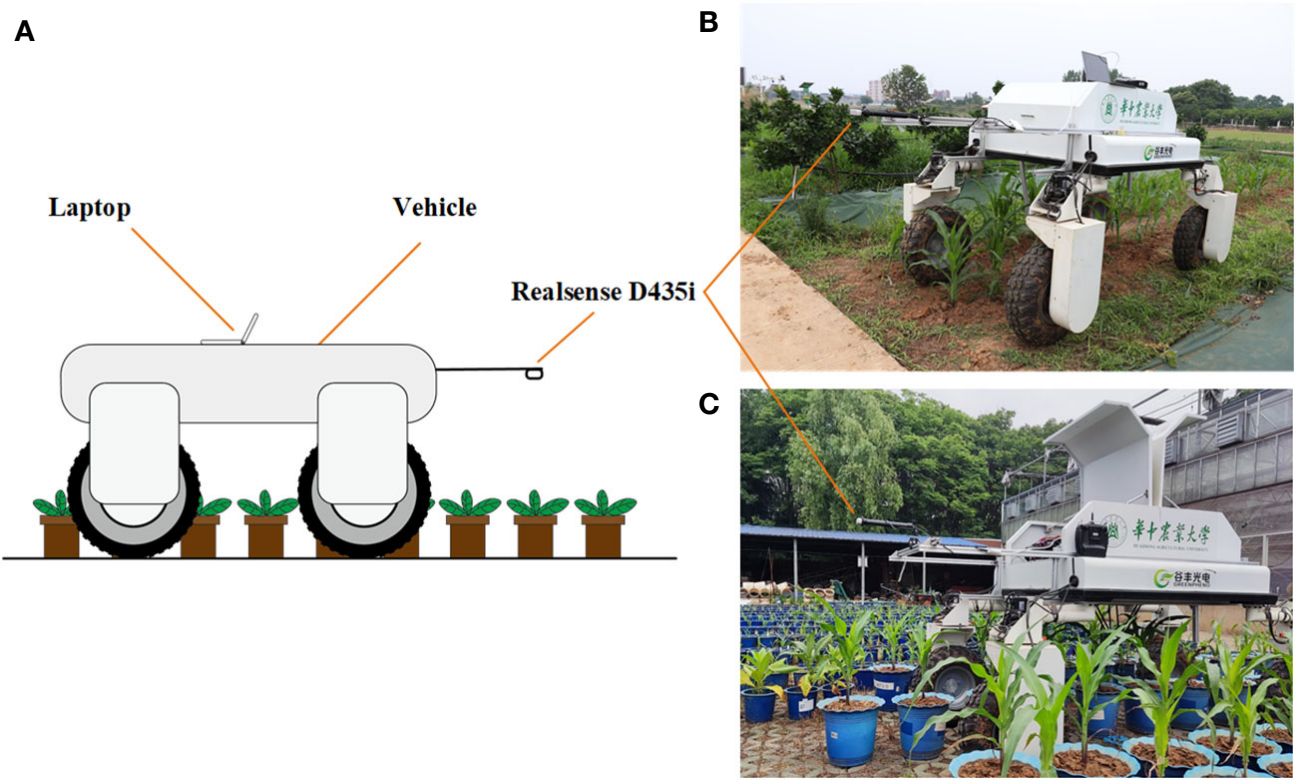
Data acquisition based on the movable platform; **(A)** Schematic diagram of data acquisition **(B)** Data acquisition in the field; **(C)** Data acquisition in pot field.

In this study, image acquisition software with the PyQt5 interface was developed, which is capable of capturing color images and depth images. The minimum working distance of the Realsense D435i camera is 0.11 m, and good depth image quality can be achieved within 1.5 m of the camera. To ensure the quality of the depth image for creating accurate 3D point clouds, the camera was placed 1.1 m away from the ground. The positive direction of the X-axis of the sensor is opposite to the direction of the platform movement, and the Z-axis of the sensor is perpendicular to the ground. The platform can move along with plant rows at different speeds during the data acquisition process. Depth images, as well as RGB images, were acquired automatically, and resolutions were both set as 1280×720 pixels at a frequency of 30 Hz in this study. The plants involved in this study were maize, tobacco, cotton and Bletilla striata. Cotton was planted in the field, while other crops were planted in pots that were managed in an outdoor environment. The movable platform was equipped with a notebook computer (Surface Pro 4 i5 8 + 256G), which was connected to the Realsense D435i depth camera and controlled the camera to acquire and store depth images and color images continuously. The data acquisition scenarios are shown in **Figure 2**.

### 2.2 Point cloud match

Point clouds of each frame from the RGB-D sensor were combined by depth image and color image. To reconstruct the point clouds of crop rows precisely and completely during the moving process, it is necessary to match the point clouds of adjacent frames. However, during the matching process between two frames, a relatively accurate coarse matching matrix must be provided for the neighboring point clouds as a starting position for fine matching to avoid fine matching falling into local optima, which will lead to matching errors (Li et al., 2020).

In this study, the sensor data acquisition frequency was set as 30 Hz, which means that when the platform moved through the crop canopy with different speeds, the number of frames acquired was different for the same plant. More frames of one plant will be captured if the platform moves at a lower speed than that of a faster speed. To improve the data processing efficiency while ensuring the matching accuracy, and make sure the method can be applied to various mobile platforms with different speeds, not all the collected frames were used for point cloud matching. Data frames for matching were selected by analyzing the collected adjacent RGB images automatically.

Frames selected for the crop row point cloud reconstruction and adjacent point cloud coarse matching matrix were both determined by the shift of the homonymous points between adjacent color images acquired by the RGB-D sensor during the moving process of the platform. To calculate the physical distance of the homonymous point offset in adjacent images accurately, the scale for calibration was photographed before data acquisition, and the conversion coefficient *I* of each pixel in the RGB image relative to the real world was calculated before the experiment by a calibration plate.

The SIFT operator was calculated separately from the original RGB images of two selected frames for matching. Descriptor sets of reference image  R_i_=(*r*_*i*1_,*r*_*i*2_,*r*_*i*3_,⋯,*r*_*i*128_) and observation image  C_i_=(*c*_*i*1_,*c*_*i*2_,*c*_*i*3_,⋯,*c*_*i*128_) were created, and the descriptors of feature points within the two sets were measured by Euclidean distance (Eq. 1). Through the experiment, feature points in the observation image with a distance less than 0.3 from the reference image are retained.


Eq.1 $$d\left(R_{i},C_{i}\right)=\sqrt{\sum_{j=1}^{128}{\left(r_{ij}-c_{ij}\right)}^{2}}$$

Due to the perspective relation in color images, each pixel shows a different scaling ratio with a different distance away from the target. Because the RGB-D camera can acquire RGB images, as well as depth images, to avoid different conversion coefficients of the detected locations in color images with different depths, it is necessary to filter the (*x*,*y*) information of the detected descriptive sub locations to keep the points in the field of view that adapt to the conversion coefficient *I* . The internal reference rotation matrix of the depth image (*H*_*ir*_ ) and color image (*H*_*rgb*_ ) and the rotation matrix R and the translation matrix T of the depth image aligned with the color image are available in the SDK of the Realsense D435i camera. Here, we use Equation 2 to make the spatial coordinates of the depth image align with the same point spatial coordinates of the color image by translation and rotation.


Eq.2 $$P_{rgb}=RP_{ir}+T$$

Eq.3 $$P_{ir}=H_{ir}^{-1}p_{ir}$$

Eq.4 $$P_{rgb}=H_{rgb}^{-1}p_{rgb}$$

Eq.5 $$p_{ir}=\left(H_{ir}^{-1}p_{rgb}-T\right)R^{-1}H_{ir}$$

*P*_*ir*_ (Eq. 3) and *P*_*rgb*_ (Eq. 4) are brought into (Eq. 2); the depth information of each pixel in the RGB image (*p*_*rgb*_ ) represented by the corresponding depth image pixel *p*_*ir*_ can be related to (Eq. 5). Then, the depth information for all similar pairwise descriptors of the RGB image was calculated. In this study, some of the frames acquired were selected to reconstruct the plant point clouds. First, the first frame was recorded with both depth images and RGB images. Then, the distance of the platform moving along the working direction away from the position of the first frame was calculated by comparing homonymous points in different images by the SIFT algorithm. When the calculated distance was in the setting range, the current frame was chosen for the reconstruction. Then, the translation rotation matrix *S* was constructed as follows.


$$\text{S}=\;\left(\;\;\begin{array}{cccc} 1 & 0 & 0 & -△x\\ 0 & 1 & 0 & -△y\\ 0 & 0 & 1 & 0\\ 0 & 0 & 0 & 1 \end{array}\;\;\;\right)$$

**Figure 3:**
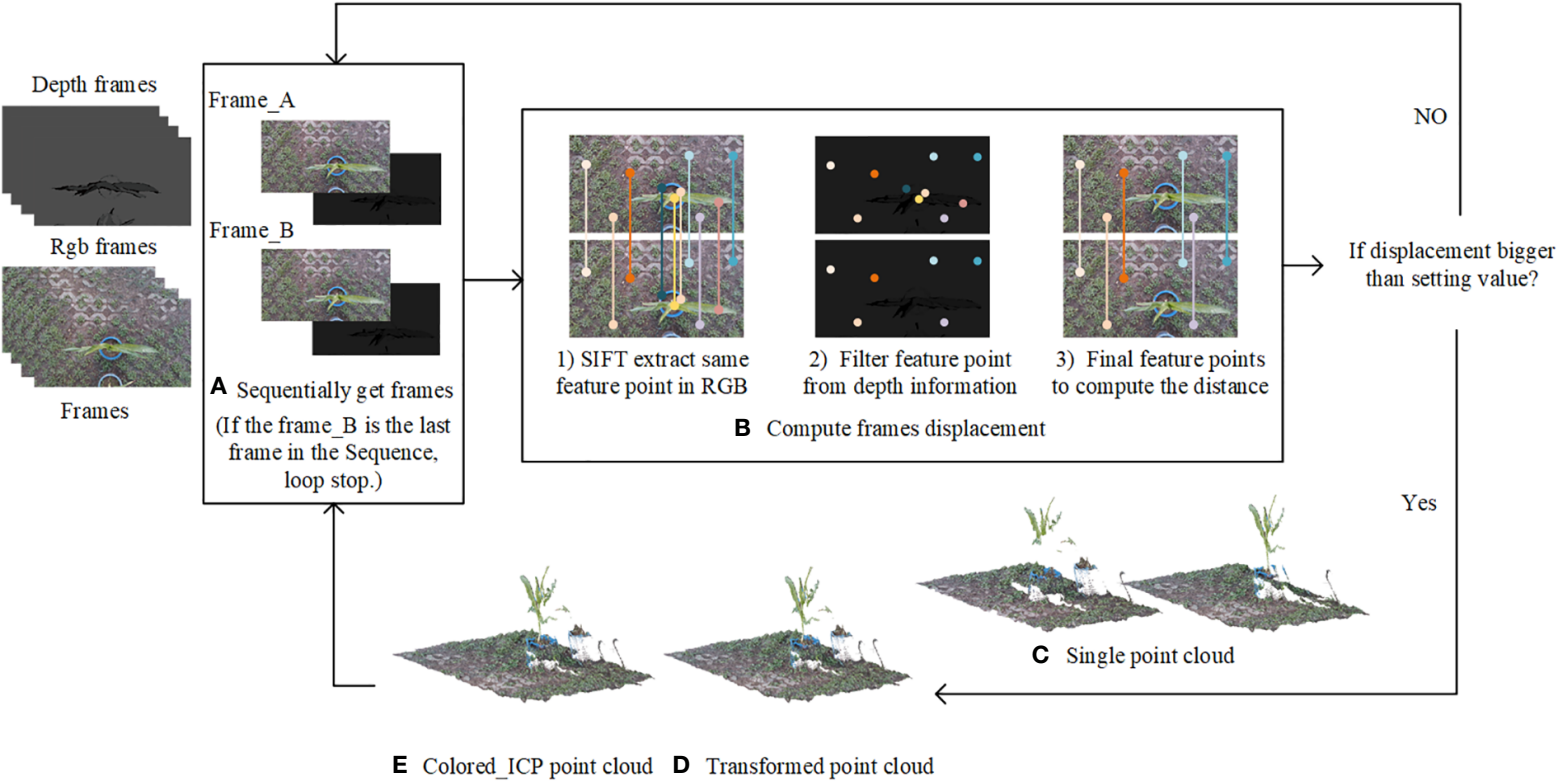
Process of crop row point cloud matching.

**Figure 4:**
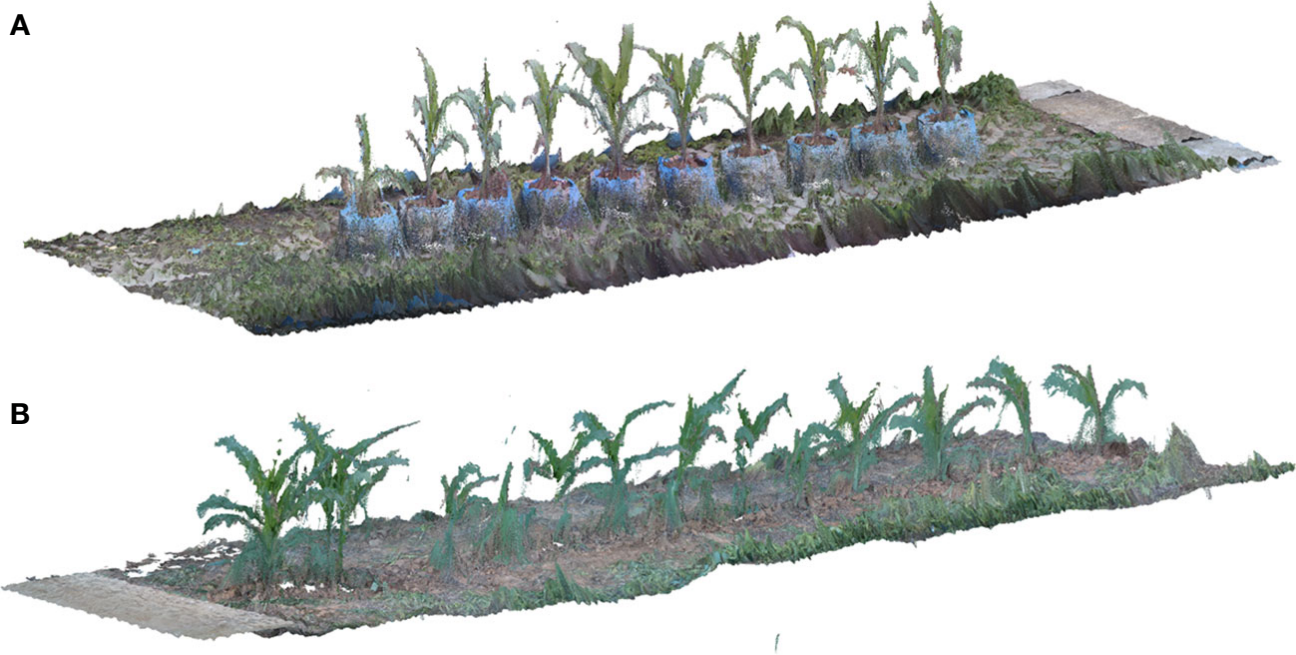
Results of crop row point cloud reconstruction **(A)** Reconstruction result of maize planted in pot **(B)** Reconstruction result of maize planted in the field.

By using the calculation method described above to obtain the depth information of each feature pixel in the RGB image, we can obtain depth information of each point in the image, and a point cloud with color can be produced after the fusion of depth information and RGB information. During the process of coarse matching, the point cloud was multiplied by the translation rotation matrix S to ensure that the point cloud had a better initial position for the fine-matching step. The colored ICP algorithm of Park et al. (Park et al., 2017), which was a derived matching scheme of ICP for reconstructing point clouds from RGB-D images, was used in the fine-matching part. The fine-matching part using the structure information and the color information from adjacent point cloud to calculate a new rotation translation matrix, to make sure the adjacent point cloud accurately merging together. The process of crop row point cloud matching is shown in **Figure 3**, and the reconstruction results of crop row in different scenarios is shown in **Figure 4**.

### 2.3 Point cloud preprocessing and individual plant segmentation

The original data of the depth sensor inevitably contain noise, and the point cloud alignment process also induces noise. Thus, preprocessing procedures are needed to remove noise from the produced point cloud. In this study, statistical filters were used to remove noise values and obvious outliers from the point cloud first. Then, a radius filter was used to define the topology of the point cloud, and the number of points within 0.002 m of each point was calculated to filter out the points with fewer than 12 neighboring points. Finally, the point cloud was packed into a 0.001 m side voxel grid by a voxel filter, and coordinate positions of the points in each voxel were averaged to obtain an accurate point (Han et al., 2017). Since plant rows were reconstructed by the proposed method, it is necessary to separate the point cloud of individual plants from the whole row data to acquire single-plant 3D phenotyping traits. Here, the clustering method was applied to segment the point cloud of each plant.

**Figure 5:**
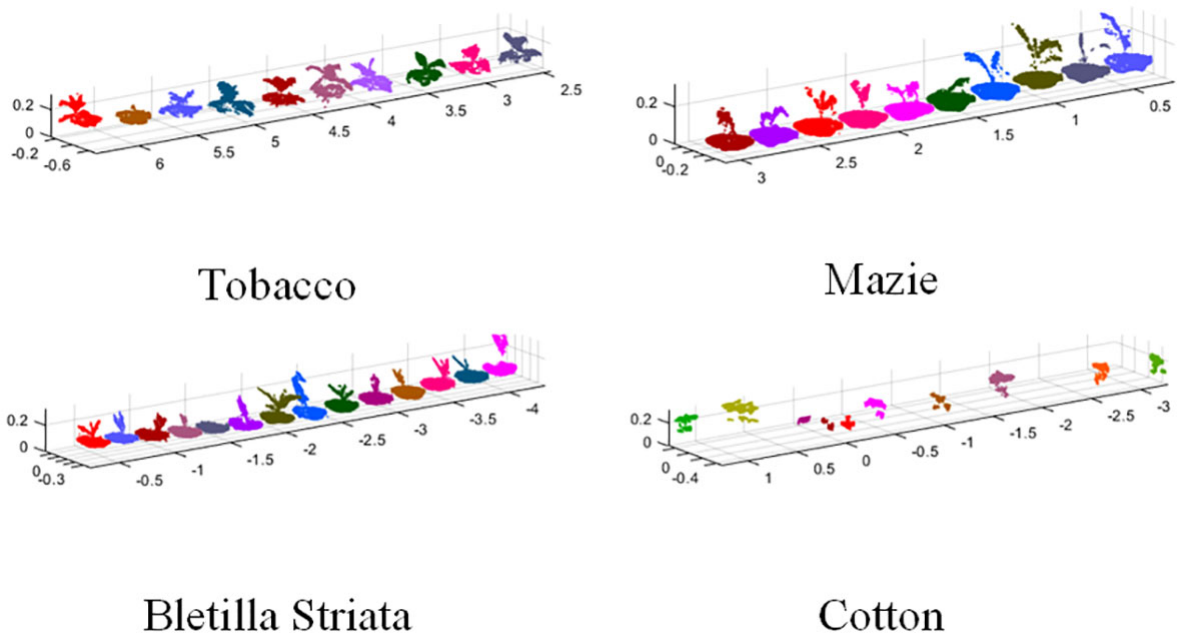
Results of point cloud preprocessing for different crops.

To cluster individual plants in the plant row point cloud, the plane of the crop row point cloud was corrected to make the plane in the point cloud parallel to the plane in the real world to accurately calculate the phenotypic traits. We used the random sampling consistency (RANSAC) method to extract the plane in the point cloud and obtain the plane equation. The normal vector of the plane in the point cloud was extracted, and the point cloud was transformed to ensure that the normal vector was parallel to the Z axis in the point cloud. Then, the downsampling method was used to speed up the point cloud clustering. We cluster the points with a minimum of 40 points and a critical distance of 0.04 m according to the density clustering method. However, the clustering method will lead to excessive plant segmentation of the point cloud. To merge the parts of a single plant cluster from the point cloud, the maximum and minimum values of XYZ in each clustered point set were saved as *C*_*i*_=[*X*_ *min*_, *X*_ *max*_, *Y*_ *min*_, *Y*_ *max*_, *Z*_ *min*_, *Z*_ *max*_] . The center of the Y axis of each cluster was calculated at the same time by *Y*_ *i*_*center*_= *Y*_ *i*_*max*_− *Y*_ *i*_*min*_ . All the clusters were compared in order if the *Y*_ *i*_*center*_ of cluster *C*_*i*_ is between the maximum value  *Y*_ *max*_ and minimum value  *Y*_ *min*_ of cluster *C*_*j*_ . This indicates that cluster *C*_*j*_ contains cluster *C*_*i*_ , and the boundary data of cluster *C*_*i*_ will be deleted. The process is repeated until the *Y*_ *i*_*center*_ value of any cluster is not within the range of maximum value  *Y*_ *max*_ and minimum value  *Y*_ *min*_ of other clusters. The point cloud of each cluster was saved, and the original point cloud was segmented according to the boundary of each cluster by [*X*_ *min*_, *X*_ *max*_, *Y*_ *min*_, *Y*_ *max*_, *Z*_ *min*_, *Z*_ *max*_]+0.002 . The segmentation result between the plant and the ground was obtained as the individual plant point cloud. Individual plant was extracted from the point cloud of the crop row according to the clustering method mentioned above, and the Otsu algorithm was used to segment the foreground point cloud and the background point cloud for plants. Then, each point in the three-dimensional point cloud was segmented by using the 2G-B-R model through point cloud color information to obtain the point cloud of the plant area. The area outside the plant was also segmented by color to obtain the point cloud data of the soil layer. The results of point cloud preprocessing for different crops outdoors are shown in **Figure 5**.

## 3 Experiments and results

### 3.1 Point cloud reconstruction with different moving speeds

**Figure 6:**
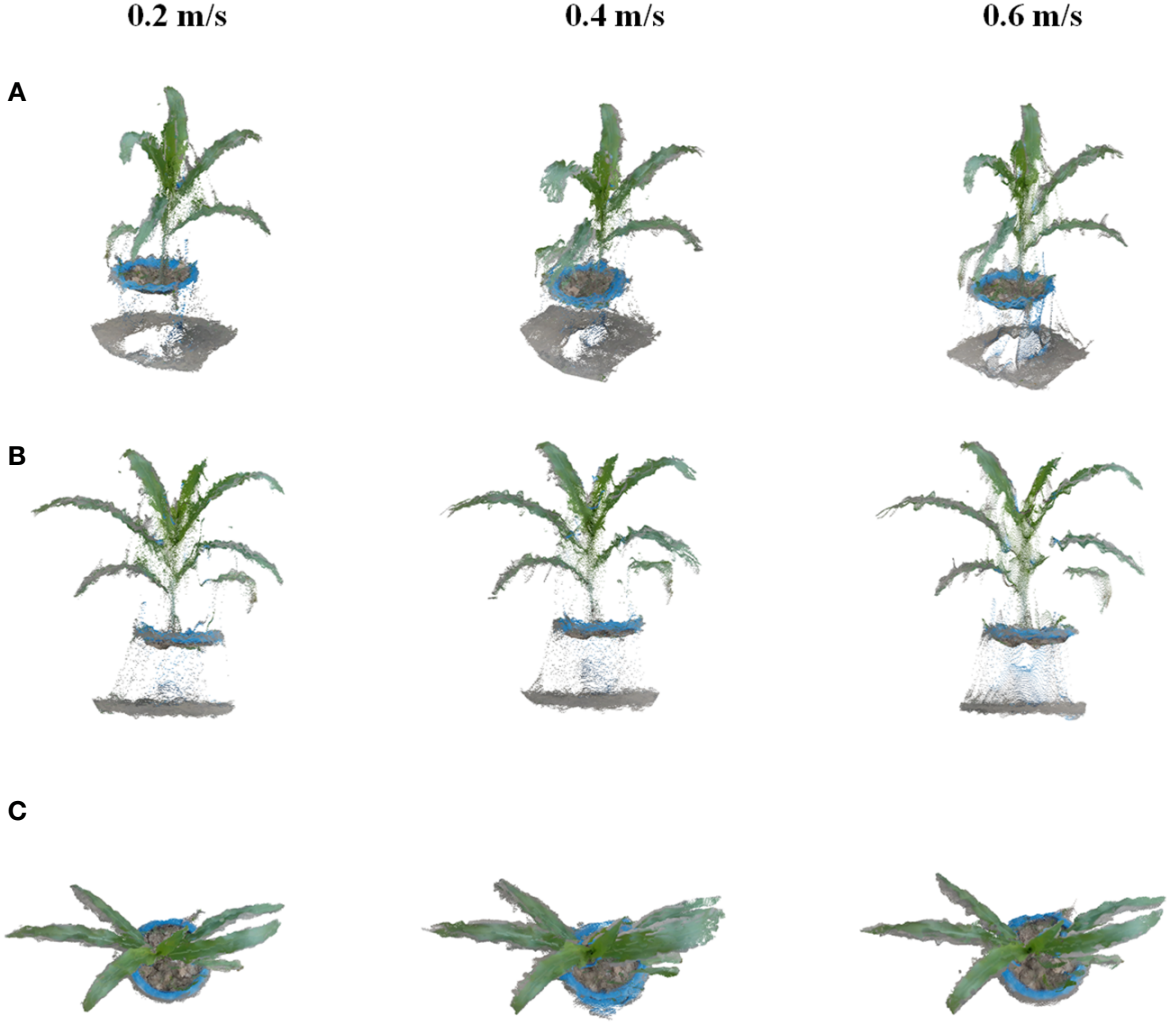
Results of dynamic reconstruction for corn at speeds of 0.2 m/s, 0.4 m/s, and 0.6 m/s. **(A–C)** are different views of single crop reconstruction in plant rows with different speeds.

Seedling plants, including corn, tobacco, cotton, and Bletilla striata, were chosen for this study. Cotton was planted in the field, while other crops were planted in pots that were managed in an outdoor environment. Experiments were conducted to verify the effects of different moving speeds of the platform by the method proposed in this study. The platform was set to three speeds of 0.2 m/s, 0.4 m/s, and 0.6 m/s, with coherent speeds for field data acquisition. The method can automatically select data frames for 3D point cloud reconstruction with different moving speeds, and the reconstruction results are shown in **Figure 6**. The results indicated that the method can stably reconstruct the point cloud from different speeds set in the experiment. The point cloud excluding the pot under the plant shows a uniform and clear state. Therefore, the reconstruction method proposed in this study has good robustness and can achieve a more complete point cloud reconstruction of the crop with different vehicle speeds, which can be used for data acquisition according to different data analysis requirements.

### 3.2 Point cloud reconstruction at different times of the day

**Figure 7:**
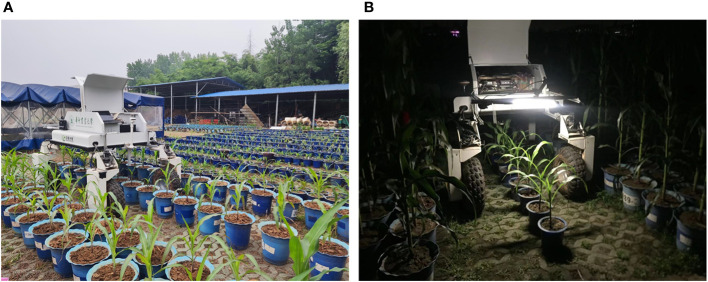
Scene of data acquisition during three periods of a day **(A)** Data acquisition in the day time **(B)** Data acquisition at night.

The moving platform was set as a speed of 0.2 m/s to investigate the robustness of date acquisition and point cloud reconstruction within different lighting environments. Since it is difficult to obtain plant color information at night, the LED light was equipped on the movable platform to obtain data at night. Potted maize and tobacco plants at the seedling stage were used as experimental objects. Data acquisition was performed in March 2022 for tobacco and in June 2022 for maize. The experiment was divided into three time periods: in the morning, in the afternoon, and at night for validation. **Figure 7** shows the data acquisition scene during different periods of the day.

**Figure 8:**
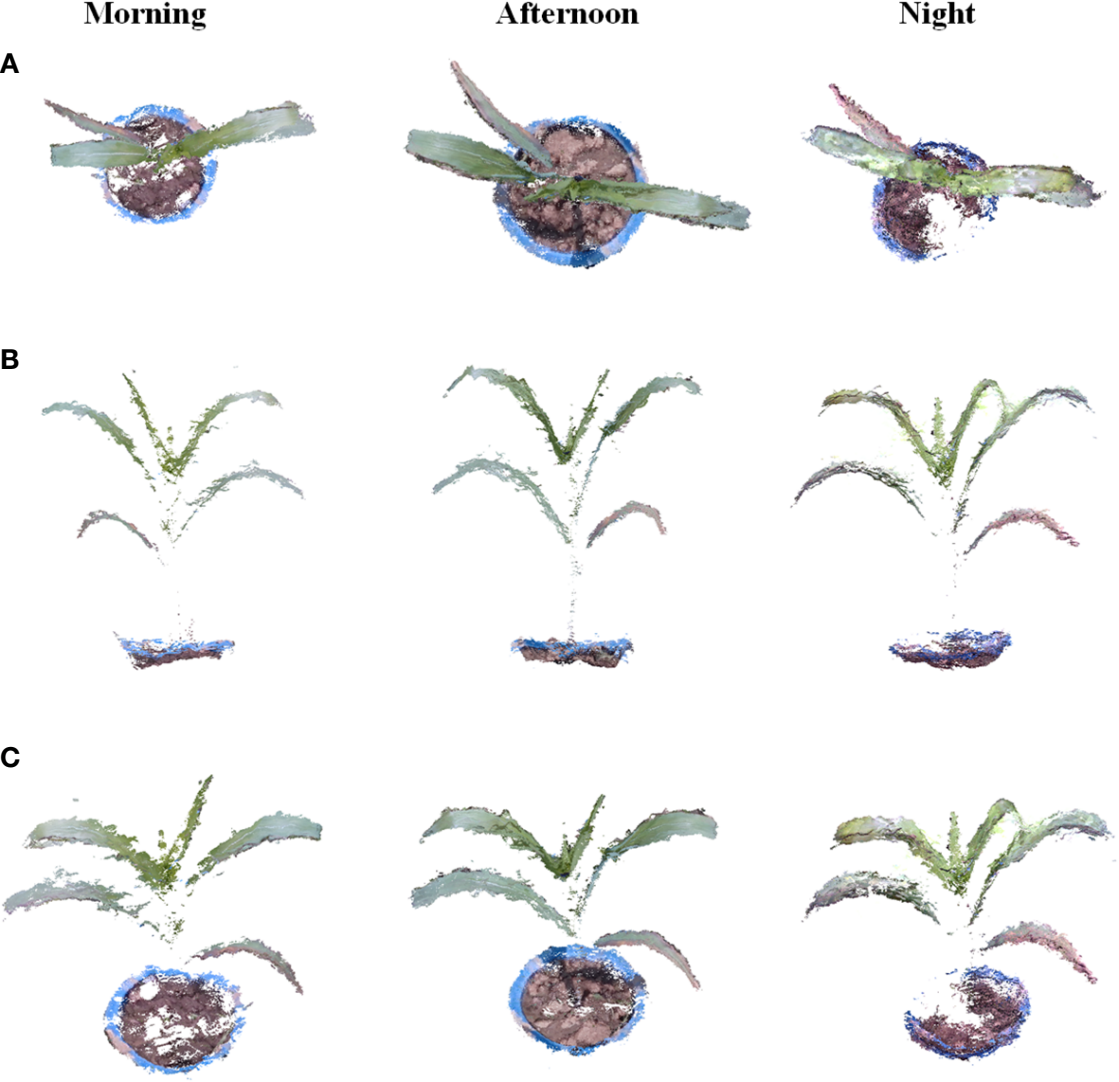
Results of the dynamic reconstruction of crops throughout the day of the experiment. **(A, B)** are different views of individual corn seedlings in the reconstructed point cloud. **(B)** shows different views of individual tobacco seedlings in the reconstructed point cloud.

As seen in **Figure 8**, the point cloud reconstructed from data captured in the morning is lighter than that of other times because the illumination in the morning is weaker than that in the afternoon, so more detailed information on plant structures, such as leaf veins and lower yellow leaves, can be shown. In the afternoon, the illumination was stronger than that in the morning, and the leaf shape was clearer than that in the morning during the reconstruction results. In the data acquisition experiment at night, it is difficult to obtain the complete detailed texture of the target in a dark environment. Therefore, the accuracy of the target depth information will be affected, and the reconstruction results were missing, especially for the part covered by leaves. Overall, the reconstruction results of the target at night were still complete, and the reconstructed plant point cloud was similar to that in the daytime.

The results show that the method we propose in this paper can obtain point clouds all day long, and the RGB-D camera shows a high potential advantage in reconstructing plant point clouds. Proper illumination is helpful for selected RGB-D cameras to acquire depth information, and the method can also achieve normal results at night.

### 3.3 Comparison of point clouds with different methods

To verify the effectiveness of the method proposed in this study, potted plants of corn and Bletilla striata were used to collect data out of the door using a LIDAR from FARO and a Canon 77D cameras, respectively. Five stations were set up to scan while using a laser beam to scan corn and Bletilla striata plants, and the SCENE software developed by FARO was used to merge multiple sites to obtain the best quality point cloud information. Images were taken by a Canon 77D camera with a lens of 18-135 mm, and the image resolution was 6000×4000 pixels. The camera was mounted on the platform to capture images of plant rows vertically and continuously downward. During image processing, the software *Visual-SfM* was used for sparse reconstruction (Zheng and Wu, 2015), and the multiview stereo (MVS) algorithm developed by Furukawa and Ponce was used for dense plant reconstruction (Furukawa and Ponce, 2010).

**Figure 9:**
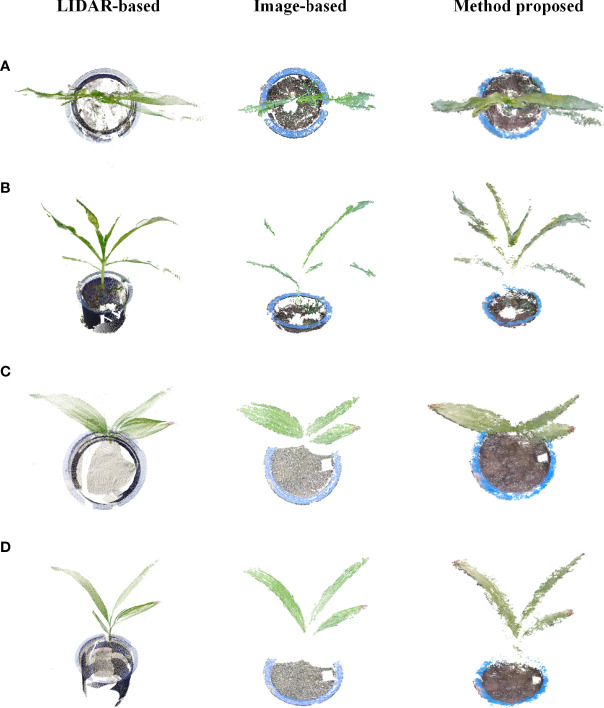
Comparison of LIDAR-based, image-based and proposed methods for 3D reconstruction. **(A, B)** are different views of corn point clouds, and **(C, D)** are different views of Bletilla striata point clouds.

As shown in **Figure 9**, the number of point clouds obtained by the 3D scanner based on LIDAR was relatively sparse in this experiment, and a certain number of point clouds can be seen missing from the top view, but the overall point cloud was relatively complete and uniform. For the image-based 3D reconstruction method, 127 images for a single Bletilla striata plant and 140 images for a single corn plant were taken for 3D reconstruction. Due to the wrinkles on the surface of maize leaves, the reconstruction effect was relatively poor, and there were also many missing details in the reconstruction of Bletilla striata. In comparison, the proposed method could obtain a dense point cloud, but its reconstruction effect was not as good as that of LIDAR. However, it can reflect more details of plants and leaves than the method of image-based reconstruction.

### 3.4 Effectiveness of phenotypic trait extraction

#### 3.4.1 Evaluation of plant height

**Figure 10:**
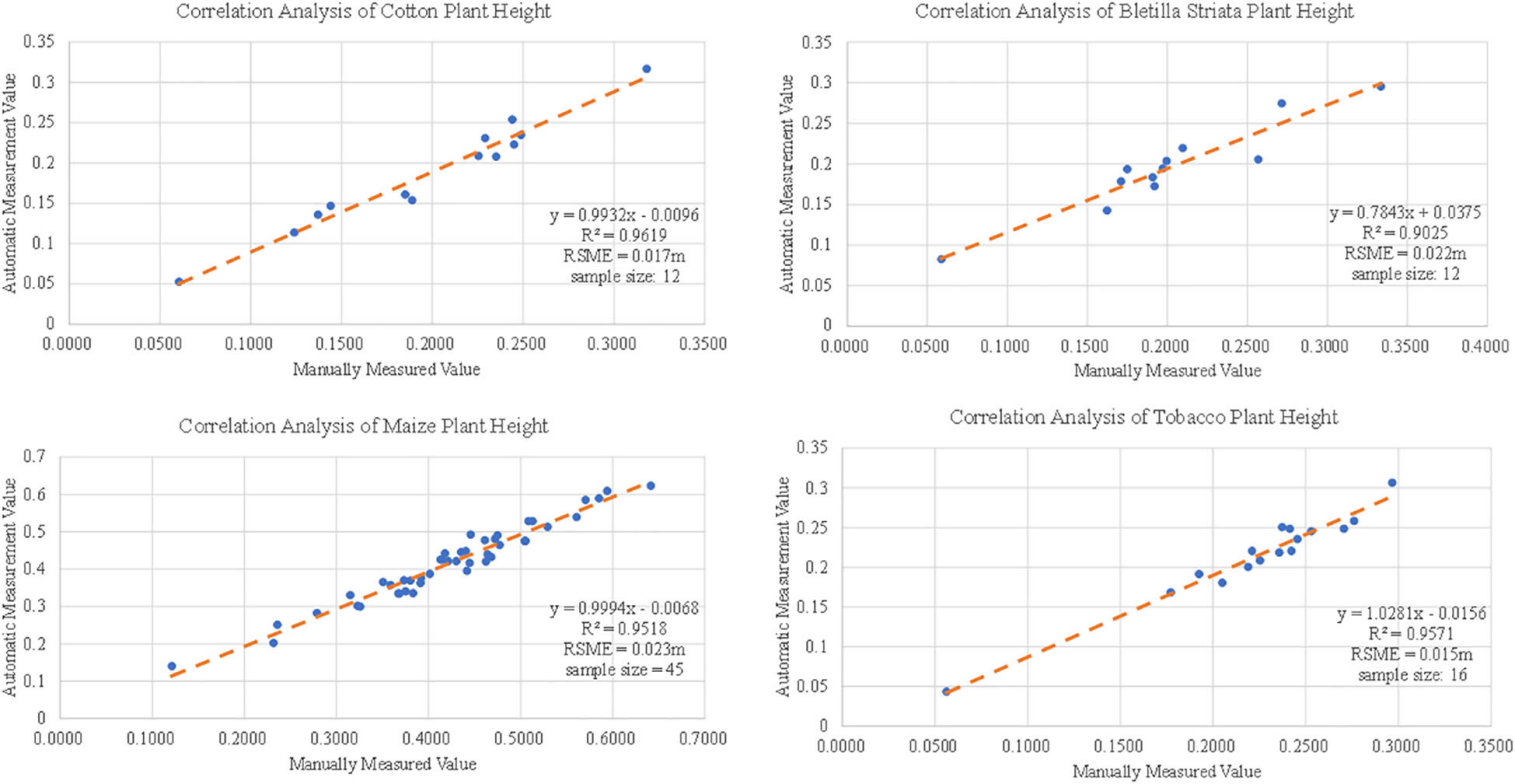
Correlation analysis of tobacco, potato, maize and Bletilla striata plant height data.

Theoretically, plant height is defined as the shortest distance between the upper boundary (the highest point) of the main photosynthetic tissues (excluding inflorescences) and the ground level (Pérez-Harguindeguy et al., 2016). The distance between the surface of the soil layer and the individual plant top was measured as the plant height in this experiment. The RANSAC method was used to fit the soil layer plane, and the distance from the highest point of the plant area to the plane was calculated as the calculated height of each plant compared with the actual value measured manually. Seedlings of tobacco, potato, corn and Bacillus striata were tested in this experiment. The results are shown in **Figure 10**. The test samples include 12 cotton, 45 corn, 16 tobacco and 12 Bletilla striata. Plant height showed strong agreement in cotton (R^2^ > 0.96), corn (R^2^ > 0.95), tobacco (R^2^ > 0.95) and Bletilla striata (R^2^ > 0.90) and with a good error index for cotton (RMSE = 0.017 m), corn (RMSE = 0.023 m), tobacco (RMSE = 0.015 m) and Bletilla striata (RMSE = 0.022).

#### 3.4.2 Evaluation of plant leaf area

**Figure 11:**
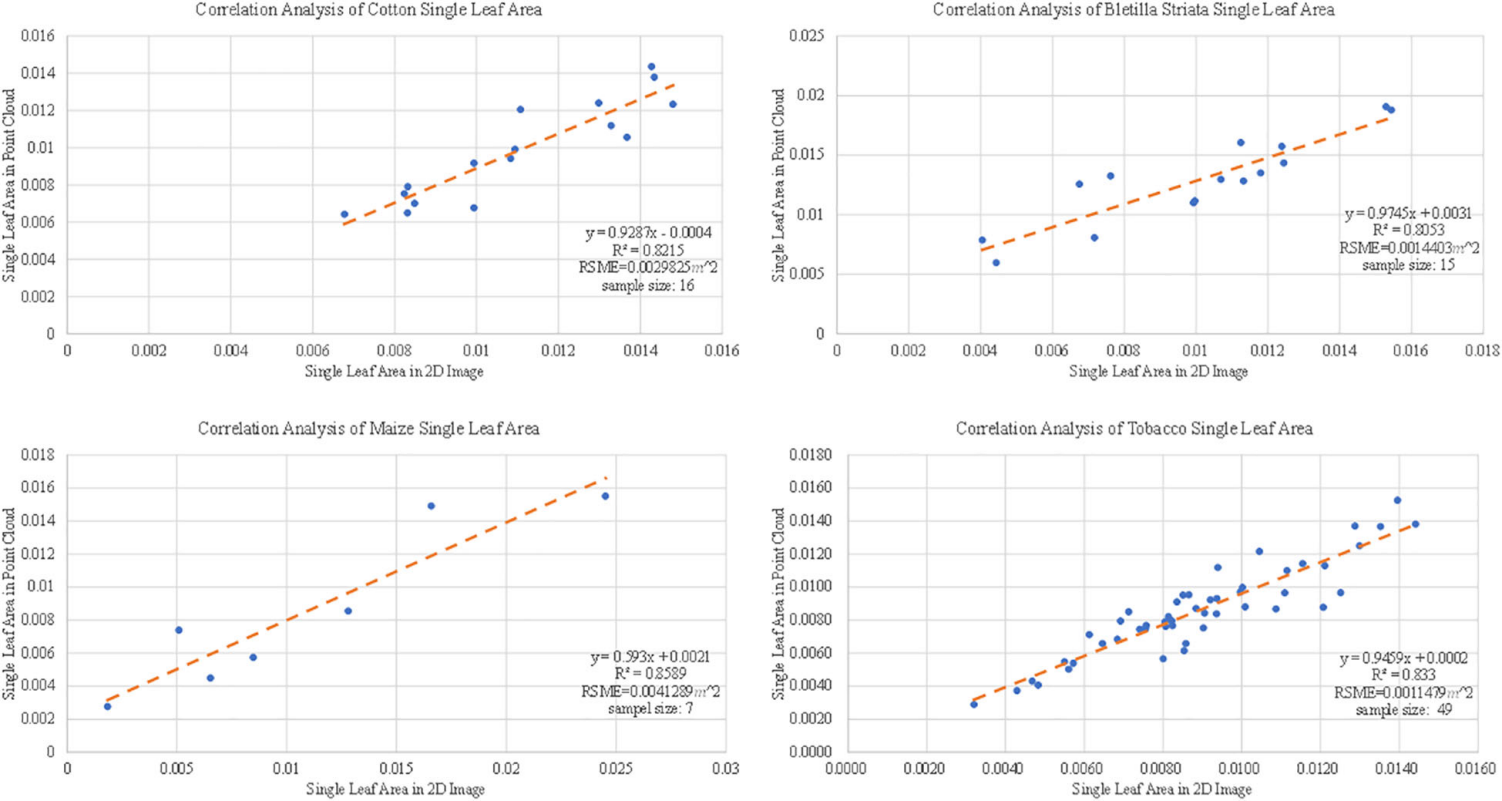
Correlation analysis of leaf area of cotton, maize, Bletilla striata and tobacco.

To verify the 3D reconstruction effect of the proposed method, the scanned crop leaves were manually removed. To get the ground truth of plant leaf area, we put leaves under a glass plate with a scaled background one-by-one to shoot the images. Processing was performed using Adobe Photoshop software to mark the leaf area, and the pixel number within the leaf area was counted to calculate real area for each leaf. In the reconstructed point cloud data, the leaf point cloud was manually clipped using CloudCompare software to obtain the area of each leaf by faceting. The test samples include 16 cottons, 7 corns, 49 tobaccos and 15 Bletilla striatas. As shown in **Figure 11**, the correlations (*R* ^2^ ) of the final data were all higher than 0.8, and the maximum root mean square error (RSME) was 0.0041 *m* ^2^ for cotton, Bletilla striata, maize and tobacco.

#### 3.4.3 Evaluation of plant projection area

**Figure 12:**
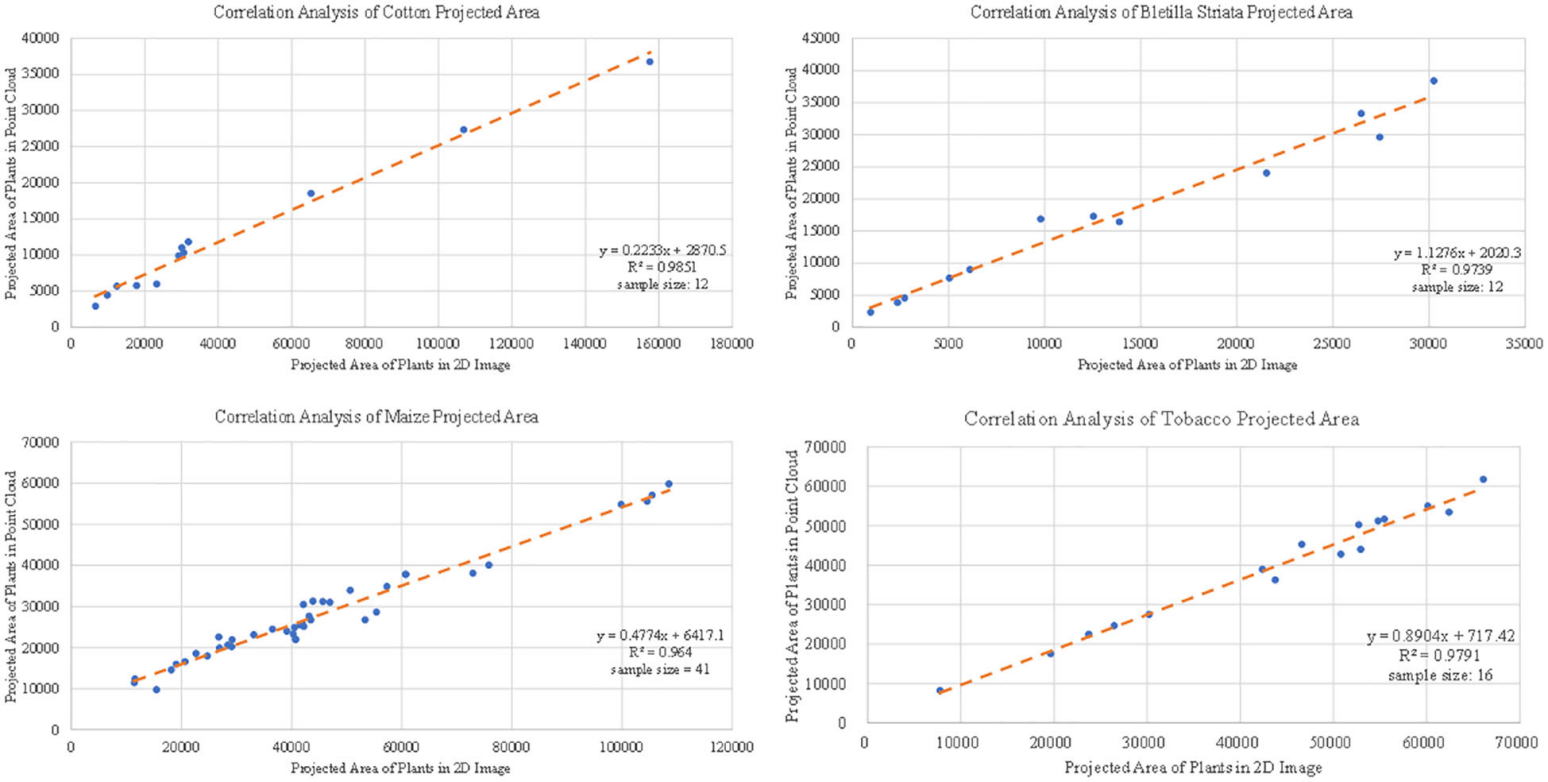
Correlation analysis of the projected area data of single crops obtained based on 3D point cloud for cotton, maize, Bletilla striata and tobacco.

The projected plant area of the canopy can reflect various growth information of plants, which can be used for plant growth monitoring (Jia et al., 2014). The 2D image was obtained by the camera above the target plant. The non-green leaf part of the plant was removed from the image using Adobe Photoshop software. In the 3D point cloud, part of the point cloud of the target plant was processed, the Z-axis information of the point cloud was deleted, a grid of 0.001 m×0.001 m was divided under the XY coordinate system, and the number of grids occupied by the point cloud was counted to quantify the projected area of the point cloud. The test samples include 12 cottons, 41 corns, 16 tobaccos and 12 Bletilla striatas. The results from 2D image and point cloud processing are shown in **Figure 12**, and the correlations (*R* ^2^ ) between the two were distributed from 0.96 to 0.99 for cotton, Bletilla striata, maize and tobacco.

## 4 Discussion

In this study, we propose a method that can reconstruct the 3D point cloud of plant seedlings with a low-cost depth camera from the plant top view by attaching it to a movable platform. The study verified the feasibility of using a Realsense D435i depth camera with different moving speeds and at different periods of a day for 3D reconstruction in outdoor environments. The accuracy of the proposed reconstruction method was demonstrated by comparing multiple acquisition device reconstruction results and individual plant features extracted with manual measurements. In the experiment, the sensor was directly equipped on the movable platform exposed to the external environment with no shading, which poses a great challenge to the sensor itself. This sensor showed good resistance to the outdoor environment, as shown in the study by Vit et al. (Vit and Shani, 2018). The proposed method provides a potential solution for continuous monitoring of phenotypic information of plant growth in all weather in the field.

In the dynamic reconstruction experiment with different speeds, we found that the reconstruction effect was affected by the crop growth stage. The results of 3D reconstruction were different when data from the same plant with the same moving speed and collection method in various growth stages were collected. The main reason for this was that the plant height and canopy projection area of crops were different at different stages. It is necessary to ensure that the sensor is approximately 0.6 m away from the crop canopy to ensure the 3D reconstruction result. Thus, the position of the sensor needs to be raised to detect a higher plant, which will cause the larger distance between the sensor and the ground to affect the accuracy of the lower part of the plant. Moreover, the lower part will be blocked by upper leaves, or data of side leaves cannot be captured completely which can be leading to the loss of point clouds. This will limit the proposed method, which is mainly applicable to crop seedlings. The problem may be solved by adding multiple sensors in the side view for data collection. In addition, a speed that is too fast will lead to insufficient frames collected for the final data reconstruction, so the maximum speed of this experiment was set as 0.6 m/s, which was restricted by the sensor performance. This problem will be resolved gradually with the advent of new types of depth sensors.

We also compared the results of plant 3D point clouds captured by LIDAR and image-based reconstruction methods. During the experiment, we found that LIDAR is suitable for large-scale 3D data acquisition of plants, but LIDAR requires much more time to scan the crop (about 10mins each scan position in our research). The disturbance of crops due to the influence of wind during the scanning process cannot be avoided. Therefore, the method has difficulty describing the point cloud of an individual plant in detail. We also used the image-based reconstruction method proposed by Jay et al. (Jay et al., 2015) for 3D reconstruction. It was found that this method can achieve high-quality data for relatively small crops (such as Bletilla striata), but for crops with relatively high plant height (such as corn seedlings), many more images are needed to reconstruct a high-quality point cloud. However, it is difficult to obtain a large number of high-quality images for a single plant in a field with intensive crop planting. Moreover, the processing of 3D reconstruction was time- and resource-consuming. The method we proposed was also inevitably affected by natural wind during the data collection; because the collection speed was faster than that of the LIDAR and image-based methods, the impact was greatly reduced.

Experiments were carried out outdoors. Compared to the indoor experiments conducted by Hu et al. using Kinect (Hu et al., 2018), the proposed method performed a lower correlation (*R* ^2^ ) and root mean square error (RSME) of the data obtained for the plant height. This is mainly because indoor environments, such as lighting and background, are controllable, which benefits the accuracy of data acquisition. In the outdoor environment, dynamic data acquisition is affected by the changing light and wind, which will cause errors between sensing data and manual measurement values. The analyzed leaf area had a lower correlation than the plant height with the actual value, which may be influenced by the different leaf attitudes and angles causing occlusion at the position of the blade root during the shooting process. Therefore, it was difficult to obtain complete point cloud data for each plant blade. Disturbances are inevitable outdoors and will also affect the results. This presents higher requirements for point cloud processing methods. A better method to process the obtained crop point cloud to judge and supplement the point cloud of incomplete leaves will improve the detection accuracy of the leaf area.

In this study, the inaccuracy of camera depth was reduced by reconstructing the point cloud from multiple viewpoints. The results are sufficient to demonstrate the feasibility of this method for high-throughput point cloud data acquisition in field environments at a low cost. It provides a reliable and cost-effective solution for breeders to acquire high-throughput plant 3D phenotyping traits in field environments and for related agricultural practitioners to acquire plant growth data.

## 5 Conclusion

This study proposes a dynamic field 3D phenotyping method suitable for various crops during the seedling stage by using a consumer-grade RGB-D camera installed on a ground-based movable platform. The results provide evidence of the method’s robustness with different moving speeds and different periods of a day for various kinds of crops. The experimental results show that (1) the image coarse matching algorithm based on the SIFT operator can provide a good initial point for fine matching, and the method can calculate the physical distance of adjacent frames, which was also effective for selecting matching frames. Combined with the colored ICP algorithm to compute a final match matrix, the whole process has shown effectiveness in the 3D reconstruction of seedling crops in the field environment. (2) The RGB-D camera-based method is suitable for various crops in the seedling stage with different moving speeds at different periods of the day. Compared with LIDAR and image-based 3D reconstruction methods, the proposed method can improve the efficiency of individual crop 3D point cloud data extraction within an acceptable accuracy, which may become a potential solution for crop 3D phenotyping outdoors. (3) The measurement of 3D phenotypic traits for corn, tobacco, potato and Bletilla striata in the seedling stage have a strong correlation with manual measurement results. The plant height (R²= 0.9~0.96, RSME = 0.015~0.023 m), leaf area (R²= 0.8~0.86, RSME = 0.0011~0.0041 *m* ^2^ ) and plant projection area (R² = 0.96~0.99). Thus, the method can be applied as a reliable and cost-effective solution for high-throughput crop 3D phenotyping in field environments.

## Data availability statement

The raw data supporting the conclusions of this article will be made available by the authors, without undue reservation.

## Author contributions

PS: conceptualization, writing original draft, review and editing. ZL: data curation, methodology, writing original draft. MY: methodology, experimental. YS: methodology, experimental. ZP: methodology, experimental. WY: funding acquisition, writing – review and editing. RZ: project administration, methodology, software, writing – review and editing. All authors contributed to the article and approved the submitted version.
